# A Case of Myxedema Coma and Adrenal Insufficiency Post Pembrolizumab

**DOI:** 10.1155/2024/5444975

**Published:** 2024-07-10

**Authors:** Andrew El Alam, Mohamad Fleifel, Dana El Masri, Bertha Maria Nassani, Jessica Abou Chaaya, Mahamadou Minkailou, Mariana Barbat, Arnaud Monier

**Affiliations:** ^1^ Endocrinology and Metabolism Division Lebanese American University Medical Center, Beirut, Lebanon; ^2^ Endocrinology and Metabolism Division American University of Beirut Medical Center, Beirut, Lebanon; ^3^ Internal Medicine and Clinical Immunology Division Hôtel Dieu de France Medical Center, Beirut, Lebanon; ^4^ Endocrinology Division Louis Pasteur Hospital, Le Coudray, France

## Abstract

**Background:**

Despite their important clinical benefits, immune checkpoint inhibitors (ICIs) are associated with a spectrum of side effects known as immune-related adverse events (irAEs). These can be of various organ system backgrounds, including dermatologic, pulmonary, gastrointestinal, and endocrine. Polyglandular endocrinopathies (PLEs) post-ICIs therapy has been reported in the literature; however, to our knowledge, only a few have been documented with pembrolizumab. *Case Report*. We present a case of a female patient who developed myxedema coma (MC) and adrenal insufficiency (AI) after 4 months of stopping pembrolizumab, a programed-cell death-1 inhibitor. The patient was clinically symptomatic and was subsequently treated with levothyroxine and hydrocortisone. *Discussion*. It is very important to be vigilant and alert in detecting MC and AI to avoid any mortality. Pembrolizumab's effect on inducing antitumor responses leads to a wide variety of multiorgan alterations. Its role in raising the risk of all-grade endocrine disorders has been previously highlighted along with thyroidal dysfunctions. Our patient's presentation falls within the literature-based median time for hypothyroidism and AI with respect to the period from the initiation of pembrolizumab. The patient's predisposition to hypothyroidism and the likelihood of intertwined manifestations of AI and hypothyroidism should always be considered in the setting of critical illness.

**Conclusion:**

It is of high significance to explore the mechanism of action of ICIs and their side effects. PLEs can house some endocrinologic emergencies that are life threatening.

## 1. Introduction

The use of immune checkpoint inhibitors (ICIs) in various malignancies has been revolutionary. Such medications have been shown to improve overall survival and, in some patients, lead to complete remission. Their use is prominent in malignancies including nonsmall cell lung cancer, malignant melanoma, renal cell cancer, and hepatocellular carcinoma [1]. ICIs include the examples of cytotoxic T-lymphocyte antigen 4 (CTLA-4) inhibitors, like ipilimumab, and programed-cell death-1 (PD-1) inhibitors, like nivolumab and pembrolizumab. With such immune-defining mechanisms of action, different autoimmune adverse effects, including endocrine ones, have been described with the use of ICIs. Symptoms and clinical signs vary depending on the target organ [2, 3]. Here, we present a case of a polyglandular endocrinopathy (PLE), manifested as myxedema coma (MC) and adrenal insufficiency (AI), following pembrolizumab therapy.

## 2. Case

A 49-year-old female patient presented to our emergency department (ED) for dysarthria, mild-to-moderate confusion, and psychomotor slowing for the past 4 hr. She had worsening fatigue and generalized edema for the past month. She had a newly diagnosed high-grade invasive ductal carcinoma and had received four cycles of chemotherapy (CTX), including paclitaxel and carboplatin, combined with immunotherapy (ITX) as pembrolizumab was used. The concluding treatment cycle was 4 months prior to this presentation, after which she underwent left-sided mastectomy. Note that the presentation was a total of approximate 8 months from the start of therapy. She is now solely receiving breast radiation therapy (RTX).

On physical exam, the patient had a blood pressure of 90−100/50−60 mmHg, corrected body temperature of 35.1°C, heart rate reaching 51 bpm, and a capillary blood glucose of 36 mg/dL. She was cooperative and could follow commands with slower than baseline responses, yet her orientation with respect to time and place GCS 11. Thyroid was palpable, nontender, and without any apparent nodules or lymph nodes. The face, mouth, and the dorsal sides of both hands and feet were all edematous and cold to touch. Cardiopulmonary and abdominal examinations were otherwise normal.

On paraclinical evaluation, an urgent brain magnetic resonance imaging (MRI) excluded any stroke or central process, plus the pituitary was intact. A transthoracic echocardiogram (TTE) showed a normal ejection fraction along with a lack of valvular heart disease. Chest X-ray was clear from any signs of infection. Labs included a normal serum creatinine of 0.83 mg/dL and within-range liver enzymes. Complete blood count and differential were all normal, and procalcitonin was negative. However, thyroid function tests (TFTs) revealed a serum thyroid-stimulating hormone (TSH) level of 73.7 mU/L (0.35–4.5), while free thyroxine (FT4) and free triiodothyronin (FT3) were both undetectable. Anti-thyroid peroxidase (Anti-TPO) antibodies reached a high of 757 U/mL (normal < 60), as thyrotropin receptor antibodies were negative.

Random serum cortisol taken on presentation plummeted at 8 nmol/L (normal 140–690), and adrenocorticotropic hormone (ACTH), while on hydrocortisone supplementation in the ED (started in ER due to suspicion of AI before taking serum cortisol level), was 2.3 pg/mL (normal 4.7–48.8). The 21-hydroxylase antibodies were negative, aldosterone was 57.0 pmol/L (61.3–979), renin 1.97 *μ*UI/mL (4.40–46.1), and a ratio of aldosterone to renin was 29 pmoL/mUI (under supraphysiologic hydrocortisone supplementation). Androstenedione and total testosterone were normal. Serum sodium was 134 *µ*mol/L and potassium was 3.7 *µ*mol/L. Liver functions AST and ALT were 44 and 58 U/L, respectively.

Based on the clinical symptoms and the paraclinical findings, the patient was diagnosed with myxedema coma (MC) and secondary adrenal insufficiency (AI), likely secondary to postpembrolizumab therapy. She was started on high-dose intravenous (IV) hydrocortisone (initially 100 mg iv bolus followed by 50 mg every 6 hr) to compensate for the severe AI, followed by levothyroxine 400 mcg IV replacement for the MC. Intense IV hydration was also applied. She received IV dextrose with external warming of 0.5°C/hr. The patient was transferred to the intensive care unit (ICU) where she was monitored closely for any signs of respiratory depression, exacerbation of further bradycardia, worsening hypoglycemia and hyponatremia, or any other hemodynamic or clinical instability.

The overall clinical status of the patient improved gradually the following day with signs of progress in her psychomotor ability and cognitive status. Her body edema also began to subside slowly. The vital signs returned to normal ranges. Follow-up TFTs on day 6 showed a significant improvement in TSH, FT4, and FT3 reaching 51.2 mU/L, 10.5 pmol/L (12–30), and 2.7 pmol/L (2–7), respectively. As a result, the supplementation of levothyroxine was switched to oral therapy (100 mcg daily). As for the hydrocortisone, the dose was switched to 10 mg by oral route twice daily. The patient was successfully discharged home a few days later after a return to neurologic and psychomotor baseline. On follow-up clinic visit, 7 weeks postdischarge, the patient was clinically well and denied any complaints. TSH, FT4, and FT3 were 11.75 mU/L, 13.5, and 4.3 pmol/L, respectively. The patient was put on levothyroxine 125 mcg daily while keeping the same dose of hydrocortisone.

Cosyntropin stimulation test failed to increase the morning serum cortisol to a normal range at 30 min (129 nmol/L) and at 60 min (103 nmol/L) (target was >500 nmol/L), which confirmed the persistence of AI. Thyroid ultrasound showed homogeneous thyroid parenchyma with hypervascularized hypoechoic areas on color Doppler that suggested foci of thyroiditis. The patient was kept on oral levothyroxine and hydrocortisone, with continued follow-up planned ahead.

## 3. Discussion

Both MC and AI are life-threatening conditions that require rapid action. AI's Addisonian crisis can reach a mortality of 6%, while MC, despite treatment, has a high mortality rate of 60% [4, 5]. The importance of a stress dose IV steroid is of high priority in patients with high suspicion for MC to avoid an adrenal crisis, regardless of the underlying adrenals' state [5]. Our case outlines a severe form of IXT-induced PLEs. Prompt action with the administration of IV steroids and later levothyroxine was key in the survival of our patient. There are different types of concurring PLEs reported by various cases in the literature using ipilimumab and nivolumab separately or in a combination therapy. Our case describes a PLE (MC and secondary AI) as a result of the usage of treatment with pembrolizumab.

Pembrolizumab binds on T-cells to block PD-1 ligands which halts the negative immune regulation, reverses T-cell suppression, and induces antitumor responses [6]. Being an ICI, pembrolizumab carries with it a spectrum of adverse reactions including endocrinologic ones (e.g., hypothyroidism, hyperthyroidism, hypophysitis, AI, and diabetes mellitus) [6]. A systematic review and meta-analysis of 12 clinical trials, that looked at the immune-associated endocrine disorders linked to PD-1 inhibitors therapy for solid tumors, suggested that pembrolizumab could raise the risk of all-grade endocrine disorders (9.85 folds), hypothyroidism (7.73 folds), and hyperthyroidism (five folds) [7].

Pembrolizumab-induced thyroid immune-related adverse events (irAEs) range between 3.2% and 10.1% [8]. Grade ≥ 3 hypothyroidism occurs in roughly 0%–2% of patients receiving combination ICIs [9]. In a study that looked at pembrolizumab-induced thyroid disease in melanoma patients, thyroid dysfunction was reported to occur within a 6-week median time (range 3–40) [10]. For the pembrolizumab-induced hypothyroidism, it was estimated around 5.7 weeks (range of 3–40) [10]. Isolated hypothyroidism was present in 6/17 patients and 9/12 thyrotoxicosis cases progressed to hypothyroidism. More than half of the hypothyroid patients necessitated levothyroxine therapy. As for thyroid antibodies, 4/10 cases had elevated antibodies with three of them being anti-TPO [10]. Our patient's presentation was approximately 32 weeks from initiation of pembrolizumab. Her last IXT was around 17 weeks prior to the ED presentation. The positive anti-TPO antibodies show that the patient likely had the genetic predisposition for chronic thyroiditis which remained dormant up until the oncology therapy triggers (CXT, RXT, and IXT). Although pembrolizumab's irAEs mechanism of action is still unclear, it is postulated that the development or activation of antithyroglobulin or anti-TPO antibodies by this selective anti-PD-1 antibody might be an underlying factor [10]. However, based on the previously mentioned literature, thyroid autoantibodies should not be affected directly by pembrolizumab, but it is the whole immunological alterations caused by T lymphocytes, natural killer cells, and/or monocyte-mediated pathways which are frequently associated with anti-PD1 antibodies [7]. Administration of an anti-CTLA-4 antibody results in both isolate ACTH deficiency and hypophysitis, while treatment with an anti-PD-1 or PD-L1 antibody only induces isolate ACTH deficiency. This could explain why our brain MRI revealed normal findings and no hypophysitis [11].

Within the irAEs domain, AI is scarce with primary AI (PAI) occurring in 0.7% of cases [12]. Another systematic review reported that pembrolizumab did not increase the risk of PAI [7]. Out of five pembrolizumab-treated patients, four developed AI with grade > 3 [12]. The median time of onset of the AI was 4.63 months from treatment, and corticosteroid treatment showed partial efficacy in four patients [12]. Our patient's apparent AI appeared around 4 months after cessation of the IXT, and persisted upon outpatient follow-up. Screening for endocrinopathies is recommended for patients receiving ICI, due to the high risk of developing immune-related adverse events. Before treatment initiation, TSH, free T4, fasting glucose, electrolytes, and early morning cortisol should be taken for every patient. ACTH levels should be added when cortisol levels are low (below 5 *µ*g/dL). Monitoring should be as such during each infusion for a minimum of 6 months, with decreasing frequency thereafter [13].

## 4. Conclusion

MC and AI are two major endocrinologic emergencies. Their co-occurrence in a patient already battling a malignancy is definitely a challenging task. Approximately 12% incidence of simultaneous hypothyroidism and AI has been previously reported in critically ill hospitalized patients [11]. Appropriate paraclinical evaluation is needed in patients planned for ICI throughout the course of ICI therapy [12]. Diagnostic vigilance and appropriate treatment applied are of high importance in order to avoid patient mortality. Continuous long-term endocrinological follow-up and evaluation are imperative, as the majority of patients do not regain their endocrine function [14].

## Data Availability

All data underlying the results are available as part of the article and no additional source data are required.
